# Editorial: The role of vitamin D in metabolic and cardiovascular health, volume II

**DOI:** 10.3389/fnut.2025.1759053

**Published:** 2026-01-07

**Authors:** Ivana Šarac, Jasmina Debeljak Martačić, Kathryn Hart, Jelena Milešević

**Affiliations:** 1 Centre of Research Excellence in Nutrition and Metabolism, Group for Nutrition and Metabolism, Institute for Medical Research, National Institute of the Republic of Serbia, University of Belgrade, Belgrade, Serbia; 2 Discipline of Nutrition, Exercise, Chronobiology and Sleep, School of Biosciences, Faculty of Health and Medical Sciences, University of Surrey, Guildford, United Kingdom

**Keywords:** chronic kidney disease (CKD), critically ill, gestational diabetes, hypertension, mortality, polycystic ovary syndrome (PCOS), thromboembolism, vitamin D

Over the past two decades, few nutrients have attracted as much scientific and public attention as vitamin D (VitD). Originally recognized almost exclusively for its pivotal role in calcium homeostasis and bone metabolism, VitD has emerged as a pleiotropic secosteroid hormone with widespread physiological functions. Advances in molecular biology, endocrinology, and immunology have profoundly expanded our understanding of VitD signaling pathways, revealing its importance in numerous tissues far beyond the skeletal system (1, 2). As a result, VitD deficiency has been increasingly implicated in a wide range of chronic non-communicable diseases, particularly those linked to metabolic health, immune function, and cardiovascular physiology (2–5).

Correspondingly, global interest in VitD has grown exponentially. This surge reflects both an increased prevalence of VitD deficiency worldwide (6–8) and the expanding body of evidence linking inadequate VitD status to obesity, insulin resistance, type 2 diabetes, metabolic syndrome (MetS), dyslipidemia, non-alcoholic fatty liver disease (NAFLD), hypertension, atherosclerosis, and cardiovascular morbidity and mortality (5, 9–15). Parallel interest emerged during the COVID-19 pandemic, when VitD deficiency was proposed as a contributing factor to increased disease severity and poor outcomes, particularly in individuals with pre-existing metabolic and cardiovascular dysfunction (16–18).

However, despite widespread enthusiasm and significant scientific advancements, controversies persist, and findings across mechanistic, epidemiological, and interventional studies remain inconsistent (3). Many of the biological pathways through which VitD influences metabolic and cardiovascular health remain poorly defined, and “cause–and–effect” relationships are often uncertain (3). Moreover, the relationship between VitD deficiency and cardiometabolic disorders appears to be bidirectional and influenced by a multitude of confounding factors, including adiposity, lifestyle behaviors, genetic background, chronic inflammation, environmental exposures, and comorbidities (3). While findings from observational studies cannot reliably establish causality and Mendelian randomization studies provide mixed results, the available RCTs vary widely in design, VitD dose, baseline deficiency status, endpoints, and follow-up duration (2, 13–15, 19). Additionally, different subgroups—based on genetics, epigenetics, ethnicity, age, adiposity, inflammation levels, microbiota composition, and comorbidities—might respond differently to VitD supplementation (20, 21).

In light of the complexities and significant scientific gaps related to the causal pathways connecting VitD to metabolic and cardiovascular health, in 2022, we established the Frontiers Research Topic titled “*The Role of Vitamin D in Metabolic and Cardiovascular Health*.” This Research Topic aimed to address these gaps by presenting studies that focused on: (1) the pathophysiological mechanisms underlying the bidirectional relationships between VitD and metabolic or cardiovascular disorders; (2) epidemiological evidence across various population subgroups; (3) individual responses to VitD supplementation and its potential as an adjunctive therapy for individuals with cardiometabolic diseases; and (4) the role of VitD as an immunomodulator (3). Due to the success of this Topic and the 13 high-quality articles associated with it, in 2024, we launched the Topic extension titled “*The Role of Vitamin D in Metabolic and Cardiovascular Health - Volume II*.”

The present volume builds on the first volume's findings and presents nine new contributions that deepen our understanding of VitD's multifaceted involvement in cardiometabolic, vascular, and reproductive health. The nine articles in this Research Topic reveal new layers of nuance—including stratified population risk, new mechanistic insights, new implications, and translational boundaries.

Several studies highlight the further cardiometabolic implications of VitD deficiency. Hung et al. extended the cardiometabolic risk profile by showing that VitD deficiency is associated with increased risk for venous thromboembolism (deep vein thrombosis and pulmonary embolism), in a dose-response fashion, pointing to potential effects on endothelial or coagulation pathways, as well as higher mortality and intensive care unit (ICU) admission. Li et al. demonstrated that hypertensive individuals with low VitD levels exhibit altered heart rate variability, renin-angiotensin-aldosterone system (RAAS) activity, and a greater need for coronary revascularization, implying impaired autonomic and endocrine regulation and higher cardiovascular burden.

Other studies collectively reinforce the idea that VitD's health effects are not isolated but interact with other lifestyle and biological factors. Cai et al. showed that the relationship between VitD, lipid status, inflammation, and mortality is highly modulated by glycemic status and gender, suggesting that genetic and metabolic background modify VitD's physiological effects. Wang et al. found that concurrent VitD deficiency and sleep disorders markedly increase cardiovascular mortality, indicating possible synergistic effects.

Another group of studies emphasizes VitD's role in disease progression and clinical prognosis. Lin et al. demonstrated that chronic kidney disease (CKD) patients with low VitD experience more major adverse kidney events (MAKE), higher mortality and hospitalization rates, underscoring potential renoprotective effects of VitD. Fu et al. found that among individuals with sarcopenia, VitD has a non-linear association with all-cause and cardiovascular mortality, suggesting threshold levels that may lower mortality. Zheng et al.'s meta-analysis in critically ill patients indicates that VitD supplementation may improve some clinical outcomes, particularly in ventilated patients, including short-term mortality, duration of ICU hospitalization, and need for mechanical ventilation. Nevertheless, the evidence remains inconsistent and of low level of evidence, highlighting the need for additional high-quality trials.

Two articles focus specifically on endocrine-metabolic disorders affecting women, and indicate the growing evidence of VitD's role in both metabolic and reproductive health in women. Song's opinion article argues for a plausible link between VitD deficiency and polycystic ovary syndrome (PCOS), emphasizing its role in reproductive and metabolic dysregulation, and advocates for further studies to elucidate the precise mechanism and therapeutic potential. Zhang et al.'s mini-review similarly identifies VitD insufficiency as a risk factor for gestational diabetes mellitus (GDM), with possible mechanisms involving insulin sensitivity, β-cell function, and inflammation, and calls for more high-quality intervention trials.

Taken together, the nine studies collectively depict VitD as a versatile physiological modulator that influences cardiovascular, metabolic, renal, endocrine, reproductive, and immune pathways. Across populations—including hypertensive individuals, CKD patients, sarcopenic adults, critically ill patients, infertile and pregnant women, and those at thrombotic risk—low VitD consistently associates with adverse outcomes. Importantly, several studies demonstrate that VitD's effects are shaped by contextual modifiers such as glycemic status, gender, sleep quality, and comorbid disease, highlighting the need for targeted, individualized supplementation strategies. However, more well-designed studies are needed to address the numerous gaps identified in the research presented.
